# Recent Technologies towards Diagnostic and Therapeutic Applications of Circulating Nucleic Acids in Colorectal Cancers

**DOI:** 10.3390/ijms25168703

**Published:** 2024-08-09

**Authors:** Jun Chung, Sophie Xiao, Yang Gao, Young Hwa Soung

**Affiliations:** Department of Pathology, Renaissance School of Medicine, Stony Brook University, Stony Brook, NY 11794, USA; jun.chung@stonybrookmedicine.edu (J.C.); sophie.xiao@stonybrook.edu (S.X.); yang.gao.1@stonybrook.edu (Y.G.)

**Keywords:** colorectal cancer (CRC), circulating DNAs, circulating RNAs, quantitative real-time PCR (q-RT-PCR), next-generation sequencing (NGS), RNA-sequencing (RNA-seq), microarray

## Abstract

Liquid biopsy has emerged as a promising noninvasive approach for colorectal cancer (CRC) management. This review focuses on technologies detecting circulating nucleic acids, specifically circulating tumor DNA (ctDNA) and circulating RNA (cfRNA), as CRC biomarkers. Recent advancements in molecular technologies have enabled sensitive and specific detection of tumor-derived genetic material in bodily fluids. These include quantitative real-time PCR, digital PCR, next-generation sequencing (NGS), and emerging nanotechnology-based methods. For ctDNA analysis, techniques such as BEAMing and droplet digital PCR offer high sensitivity in detecting rare mutant alleles, while NGS approaches provide comprehensive genomic profiling. cfRNA detection primarily utilizes qRT-PCR arrays, microarray platforms, and RNA sequencing for profiling circulating microRNAs and discovering novel RNA biomarkers. These technologies show potential in early CRC detection, treatment response monitoring, minimal residual disease assessment, and tumor evolution tracking. However, challenges remain in standardizing procedures, optimizing detection limits, and establishing clinical utility across disease stages. This review summarizes current circulating nucleic acid detection technologies, their CRC applications, and discusses future directions for clinical implementation.

## 1. Introduction

Colorectal cancer (CRC) is the third and second most diagnosed cancer in men and women, respectively, with approximately 1.93 million new cases in 2020 and is the second leading cause of cancer deaths globally [1,2,3]. According to the GLOBOCAN 2020 data, new cases are expected to reach 3.2 million, and the mortality rate will increase by 60% by 2040 [1,2,3]. The high mortality rate is mostly due to late detection. Because most CRC patients are asymptomatic until the cancer reaches an advanced stage, nearly half of CRC cases are usually diagnosed at a late stage, which reduces the overall survival rate. Fortunately, however, CRC is curable and preventable if detected in its early stages. This is well proven by the fact that the 5-year survival rate after primary diagnosis is 90% in the early stage compared to 13% in the late stage (stage IV) [1,2,3,4]. The overall survival rate of CRC may depend on accurate diagnosis at different clinical stages. Today, the main goal of studies is to develop tools to accurately detect CRC tumors at an early stage. CRC screening modalities currently available for diagnosis and prevention are divided into noninvasive methods including stool, blood-based, and radiologic tests, and invasive methods including flexible sigmoidoscopy (FS) and colonoscopy [5]. Although invasive tests such as tissue biopsy are considered the gold standard for diagnosis with high sensitivity, they still have significant disadvantages such as painful injury, technical difficulties related to tumor location, high cost, and low participation rate [5]. Conversely, noninvasive tests based on carcinoembryonic antigen (CEA) and carbohydrate antigen (CA19-9), commonly used as clinical biomarkers, suffer from low sensitivity and a high rate of false positive/negative results [6,7]. Each method has its own limitations. Therefore, simple, cost-effective, and accurate methods with highly sensitive noninvasive biomarkers are urgently needed to screen and diagnose CRC at an early and curable stage. 

Many researchers have put much effort into the discovery of new reliable biomarkers for early detection of CRC to improve the survival rate of patients. Over the past years, liquid biopsy as a new diagnostic concept has gained much attention. Liquid biopsy is a noninvasive approach to detect tumor-derived circulating biomarkers in body fluids by analyzing circulating cell-free DNA (cfDNA), circulating tumor-derived DNA (ctDNA), circulating tumor cells (CTCs), RNA, exosomes, and protein molecules [8,9,10]. Analysis of these components released into body fluids provides information to better understand tumor heterogeneity and more accurately predict tumor recurrence, metastasis, or treatment response through real-time monitoring and repeated testing [10]. With the development of cell isolation technology and gene detection technology, these components as novel circulating biomarkers are being extensively studied for their role in CRC and potential clinical applications. Liquid biopsy with circulating biomarkers is currently being used to monitor cancer progression and guide treatment. However, the clinical application of biomarkers for CRC diagnosis is still limited by a lack of protocol standardization to specifically isolate, sensitively detect, and accurately analyze these biomarkers.

In this review, we aim to summarize the most up-to-date methodologies used to detect circulating biomarkers in liquid biopsy and their potential clinical utility for the diagnosis and prognosis of CRC. In particular, we focus on DNA- or RNA-based circulating biomarkers associated with CRC.

## 2. Circulating Nucleic Acid in Liquid Biopsy

Among the main clinical biomarkers targeted in liquid biopsy, circulating DNA and RNA are freely available throughout blood-based biofluids [11]. Today, alterations in these biomarkers, such as abnormal expression and mutations found in CRC patients, can be easily detected by several techniques based on molecular biology. Detected alterations have been shown to correlate with tumor burden and serve as early indicators of tumorigenesis, recurrence, and drug response. Additionally, microRNAs loaded into exosomes (Exo-miRNAs) are considered to have highly valuable information not only for early diagnosis but also for advanced diagnosis, due to the fact that exosomes are capable of representing their cells of origin at metastatic sites. Numerous researchers have highlighted their potential as specific biomarkers for CRC.

### 2.1. Circulating DNAs

Circulating cell-free DNAs (cfDNA) released into blood through active processes such as apoptosis, necrosis, or active secretion, and passive processes such as inflammation, cell lysis, or tumor activity [12]. The difference in cfDNA levels between CRC groups and healthy groups has been considered an important biomarker for CRC screening tools in several studies [13,14]. However, nonspecific levels of cfDNA were observed in healthy persons or those with benign lesions, leading to false-positive results [15]. The nonspecific presence of cfDNA indicates that tissue is under stress conditions such as inflammation, tissue injury, or exercise. Hence, instead of cfDNA testing, circulating tumor-derived DNA (ctDNA) has emerged as a new biomarker for CRC detection. Unlike cfDNA, ctDNA (<1%) is a small fraction of cfDNA, originating only from tumors. Early studies have focused on detecting ctDNA levels in CRC. ctDNA is more detectable in patients with CRC than in healthy persons, suggesting that ctDNA can be used as an indicator for early diagnosis of CRC. ctDNA levels have been shown to correlate with CRC stage and tumor size, where stage IV patients had higher concentrated ctDNA than those with stage I [16,17]. High tumor recurrence rates and poor prognosis have been reported in CRC patients with high levels of ctDNA. Monitoring ctDNA levels can also predict objective response, progression-free survival (PFS), and overall survival (OS) in metastatic CRC patients after chemotherapy [18,19]. In recent years, the fact that ctDNA carries tumor-specific genetic variations (single nucleotide polymorphisms and mutations) and epigenetic modifications (methylation) consistent with the intra-tumoral parent tissue has attracted considerable interest as specific biomarkers for CRC monitoring. Numerous studies reported genetic and epigenetic abnormalities in ctDNA, suggesting the potential clinical utility of ctDNA in liquid biopsy [20,21]. Currently, significant mutations of KRAS and BRAF in ctDNA have been widely used in CRC diagnosis tests [22,23,24]. In addition, the methylation of SEPT9, TAC1, and IGFBP3 in early CRC has also shown diagnostic and prognostic value in CRC [25,26]. Indeed, this suggests that detecting aberrations of tumor markers in ctDNA at the early stage of CRC based on liquid biopsy is an effective strategy to reduce patient mortality and increase overall survival. However, most of these biomarkers are still not clinically applicable due to a lack of validation studies.

#### 2.1.1. Method for Detection of Circulating DNAs

ctDNA inherited from a tumor origin possesses excellent sensitivity, specificity, and predictive accuracy in the diagnosis and monitoring of patients’ response. However, since ctDNA represents only a very small proportion of total cfDNA, targeting and detecting ctDNA in liquid biopsies requires highly sensitive and reliable detection methods. Several technologies based on molecular biology have been adopted to track, detect, and monitor genetic alterations of circulating ctDNA in liquid biopsy samples. The current standard technologies for analyzing ctDNA are divided into PCR-based methods, such as digital PCR (dPCR), and next-generation sequencing (NGS)-based methods paired with bioinformatics analysis [27,28].

PCR-based methods are cost-effective and highly sensitive tools capable of detecting mutations even in limited amounts of input DNA. In recent years, new technologies such as droplet digital PCR (ddPCR) and bead emulsification amplification and magnetics (BEAMing) have been developed to reduce costs, errors, and background noise. ddPCR allows the detection of low variant allele frequency (VAF) by amplifying single DNA molecules. BEAMing, as a high-throughput version of PCR-based technologies, is the first clinically validated liquid biopsy test among PCR-based methods, with high sensitivity of up to 0.001%. Its commercial platforms, such as the Idylla™ system (Biocartis, Mechelen, Belgium) and OncoBeamTM RAS CRC assay (Sysmex Inostics; E.U. approval), are currently available for detecting actionable mutations in CRC. 

Based on BEAMing with flow cytometry, the OncoBeamTM RAS CRC assay was applied to detect circulating RAS ctDNA mutations in the plasma of metastatic colon cancer (mCRC) patients [22,29]. RAS mutations in plasma samples showed 93% overall concordance with tissue samples. The OncoBeamTM RAS CRC assay monitors the emergence of RAS mutations in mCRC patients treated with anti-EGFR therapy [22,30] (Table 1). This is used in routine clinical practice to determine the baseline diagnosis for selecting candidate patients for anti-EGFR therapy. These technologies have shown superior clinical applicability in ctDNA analysis. However, PCR-based methods remain limited in that they can only detect known variants at a few loci.

In comparison to the PCR-based method, the NGS-based method is designed to simultaneously detect multiple genetic alterations and unknown variants by employing high-throughput deep sequencing of multiple gene fragments at once [39]. Somatic single nucleotide variants (SNVs), variant allele frequencies (VAFs), copy number aberrations (CNAs), or DNA methylation patterns can be achieved by the NGS-based ctDNA assay [39,40]. More recent studies have reported expanded NGS applications for microsatellite instability (MSI) detection with the ctDNA fraction [41]. NGS-based algorithms, such as blood MSI signature enrichment analysis (bMSISEA), can be applied to detect MSI-H status in ctDNA isolated from blood samples of CRC patients using the ColonCore panel consisting of 41 CRC-related genes [41].

Although NGS is a reliable method for collecting genetic variant data, detecting true variants at such low variant allele frequencies (VAFs) using standard NGS techniques presents a significant challenge due to random errors during library preparation or sequencing. In early-stage clinical applications, distinguishing true mutations and avoiding mistakes from various sources of error is crucial. To overcome this technical issue, strategies utilizing unique molecular identifiers (UMIs) or unique barcodes have been recently adopted to help reduce false-negative results and increase detection sensitivity in NGS-based assays [42,43]. Furthermore, a key advantage of NGS is the ability to use a wide range of markers in a single panel, allowing for deep sequencing of target regions, genome-wide sequencing, high throughput, reproducibility, and speed. In recent years, NGS assays have been developed with targeted panels, including Tagged-Amplicon deep sequencing (Tam-seq), Safe-sequencing system (Safe-SeqS), CAncer Personalized Profiling by deep sequencing (CAPP-Seq), Integrated digital error suppression (iDES), and Ion Torrent [39,44]. NGS assays with untargeted panels include Whole-Genome Sequencing (WGS) and Whole-Exome Sequencing (WES) [44].

Targeted panels can detect point mutations and indel (insertion-deletion mutations) analysis, while untargeted panels allow the detection of clinically significant genome-wide DNA variations without needing information about the primary tumor. WGS assays have been developed with new technologies such as personalized analysis of rearranged ends (PARE), digital karyotyping, and the Fast Aneuploidy Screening Test-Sequencing System (FAST-SeqS) [39,45]. However, applying WGS is only feasible with high input sample volumes. Despite the benefits, NGS methods have limitations, such as relatively low sensitivity, high cost, and low levels of DNA in the blood. To address these limitations, NGS-based technologies have been enhanced in terms of sensitivity, reliability, and cost. Currently, the Guardant 360 assay (Guardant Health, Inc., Redwood City, CA, USA) [46] and FoundationOne Liquid CDx (Foundation Medicine, Cambridge, MA, USA) [47] are commercially available as FDA-approved liquid biopsy tests used to determine clinical trial options for patients [32,33,34,35] (Table 1).

In conclusion, there is no doubt that NGS opens new opportunities for feasible clinical applications. The detection method for circulating DNA should be chosen according to sample conditions, maximum sample throughput, purpose of analysis, and total cost. For extensive clinical utility, ctDNA tests should be developed through several clinical trial validations with acceptable sensitivity and specificity. The following are FDA-approved assays found in the market for CRC diagnosis.

#### 2.1.2. Clinical Application of Circulating DNAs

The Epi proColon test, known as mSEPT9, is the first ctDNA-based test approved by the FDA for colorectal cancer screening [48]. SEPT9 (Tumor suppressor gene septin-9) has been known as a methylation marker in CRC pathogenesis. This test is designed to analyze the SEPT9 promoter methylation status using a real-time PCR method. There are four steps: circulating DNA extraction from plasma, bisulfite conversion of DNA, purification of bis-DNA, and real-time PCR [48]. Recently, it has been developed into a second generation, Epi proColon 2.0 CE (Epigenomics AG, Berlin, Germany), to increase sensitivity and specificity and to reduce overall processing time and sample volumes [49]. Overall, the Epi proColon test showed good detection sensitivity and specificity for early-stage CRC compared to conventional noninvasive tests, including the fecal immunochemical test (FIT), blood-based CEA test, and guaiac-based fecal occult blood (gFOBT) test [31,49]. Analysis of methylation-based biomarkers in plasma ctDNA could be a promising approach for the early diagnosis of CRC, which could serve as a screening option for patients who refuse colonoscopy.

Monitoring tumor responses after surgical resection or during the course of treatment is essential to reduce the potential for micrometastases. The ctDNA analysis in blood samples of patients after surgery allows detection of minimal residual disease (MRD), which reduces the risk of recurrence and unnecessary chemotherapy [50]. ctDNA-based MRD detection in CRC is currently available through Signatera™ MRD (Natera, Inc), approved by the FDA and receiving Medicare coverage in the United States. The Signatera™ MRD test is a custom-built ctDNA monitoring assay for detection of patient-specific somatic mutations in blood samples using personalized targets found in primary tumors. This test for Stage I-IV CRC tracks 16 unique somatic mutations identified via whole-exome sequencing of an individual’s tumor using a bespoke multiplex PCR NGS method [36]. In a recent clinical study, the Signatera™ MRD test has advanced to a large platform, called CIRCURATE-Japan. ctDNA MRD data collected via CIRCURATE-Japan, comprising a large-scale patient screening registry (GALAXY) and two ctDNA-guided phase III trials (VEGA and ALTAIR), could be used as guidelines for more precise adjuvant therapy treatment regimens in patients with resectable CRC [37,38]. This platform is currently undergoing evaluation to assess the utility of ctDNA. As another ctDNA-based MRD test for CRC in the US market, Colvera™ (CRISO), launched in 2017, is available as a lab-developed test (LDT) and has Medicare coverage for MRD detection and recurrence monitoring in CRC after primary treatment [51]. It uses a real-time PCR-based method to detect methylated BCAT1 and IKZF1 in ctDNA.

Adenomas are benign tumors that can develop into malignant carcinomas if not detected and treated early. Detecting adenomas at a pre-tumor stage can significantly improve patient outcomes by enabling timely intervention. One of the significant achievements in ctDNA research is the ability to detect adenomas in pre-tumor stages. For instance, the methylation of the SEPT9 gene (mSEPT9) has been shown to be a promising biomarker for the early detection of CRC. Systematic reviews and meta-analyses have reported that mSEPT9 tests exhibit high sensitivity and specificity for early-stage CRC detection [52]. This test can identify CRC at a very early stage, which is crucial for improving patient outcomes through timely intervention and treatment. Similarly, the SDC2 gene methylation has been validated as an effective biomarker for detecting colorectal adenomas and early-stage CRC. A meta-analysis revealed that the SDC2 methylation test has a pooled sensitivity of 81% and a specificity of 95% for CRC detection. For adenomas specifically, the sensitivity was reported to be around 47%, indicating its potential for early detection before the adenomas progress to malignant stages [53].

Together with advanced new detection technology, ctDNA-based liquid biopsies have been considered a promising approach for CRC patient management. However, it is still too early to claim it as a prime test in the clinic due to the limited amount of ctDNA present in the plasma of patients with early-stage cancer.

## 3. Circulating RNAs

Circulating RNAs (circulating cell-free RNAs, cfRNAs) are mainly represented by microRNA (miRNAs), long noncoding RNA (lncRNAs), and messenger RNA (mRNAs). cfRNA is released into the blood through mechanisms such as apoptosis, microvesicle shedding, and exosome signaling [54]. Since cfRNAs regulate tumor-related transcripts, the quality and quantity changes of cfRNAs have recently attracted considerable attention as specific biomarkers related to cancer progression. Among cfRNAs, circulating microRNAs (miRNAs) have been a major focus of cfRNA studies, and numerous researchers have highlighted the potential of circulating miRNAs as a new generation of biomarkers for diagnosis, prognosis, and therapeutic prediction [55]. They have attracted more attention due to the fact that circulating miRNAs are relatively abundant, remarkably stable against endogenous RNases in RNase-rich blood, and insensitive to pH changes and temperature [55,56]. Therefore, unlike cfDNA, which requires at least 2 mL of blood for detection, circulating miRNA are detectable with just 200 ul of plasma due to their stability. The stability in serum and plasma could be explained by protective mechanisms such as miRNA modification, miRNA-binding protein complexes [57], lipoproteins (or platelets) [58], or encapsulation into extracellular vesicles (EVs) [56,59]. Most interestingly, the expression pattern of circulating miRNAs is correlated with the degree of tumor progression, which indicates their potential as noninvasive biomarkers to detect tumors at different stages. However, the isolation, measurement, and detection of circulating miRNAs remain a challenging field. Here, we summarize the current state of the miRNA field and highlight new innovative technologies, which suggest future directions of investigation for clinical application in CRC.

### 3.1. Current State of Circulating miRNAs: Detection Method and Challenges

The presence of circulating miRNAs in blood and their potential as cancer markers was first reported by Lawrie et al. in 2008 [60]. The authors demonstrated that high levels of specific circulating miRNAs are associated with clinical outcomes in diffuse large B-cell lymphoma patients. Since then, numerous studies have reported that circulating miRNAs have the ability to discriminate between healthy individuals and cancer patients [61]. In addition, several miRNAs have been shown to have specific signatures that reflect disease state and cancer progression [62]. These studies open up the possibility of applying circulating miRNAs as diagnostic and prognostic biomarkers in clinical trials. Ongoing research is aimed at isolating and detecting specific circulating miRNAs in body fluids.

The common method to detect circulating miRNAs comprises three main steps: RNA extraction, reverse transcription, and miRNA quantification. Small RNAs are isolated from different components including whole blood, coagulation factors, proteins, lipoproteins, and exosomes by using commercial extraction kits such as Trizol (Ambion, Austin, TX, USA), QIAzol (Qiagen, Hilden, Germany), mirVana PARIS kit (Ambion, Austin, TX, USA), miRNeasy serum/plasma kit (Qiagen, Hilden, Germany), and miRCURY (Exiqon, Woburn, MA, USA). Some studies have reported a comparative evaluation of commercial RNA extraction kits from serum [63], but it is still difficult to choose the ideal miRNA extraction kit for serum/plasma due to many variables, such as initial fluid volume and sample stability. In addition, there are some technical challenges. The first concern is that RNA extraction methods lack specificity to isolate only miRNAs [64,65]. As such, their concentration is always overestimated due to the presence of a mixture of small and large RNAs or hemolysis. Another concern is that the yield of miRNAs isolated from serum/plasma is too low to quantify accurately [64,65]. This causes efficiency, accuracy, and reproducibility issues in serum/plasma miRNA analysis. Therefore, the expression results of circulating miRNAs may vary depending on experimental settings such as yield and quality. The use of NanoQuant (Tecan Infinite^®^ 200 PRO, Tecan, Maennedorf, Switzerland), Nanodrop 2000 (Thermo Scientific TM, Waltham, MA, USA), or a Qubit^®^ 2.0 Fluorometer (Life Technologies, Carlsbad, CA, USA) is essential for obtaining valid results. Optimized standard protocols for sample preparation are needed for successful miRNA expression profiling.

**Figure 1:**
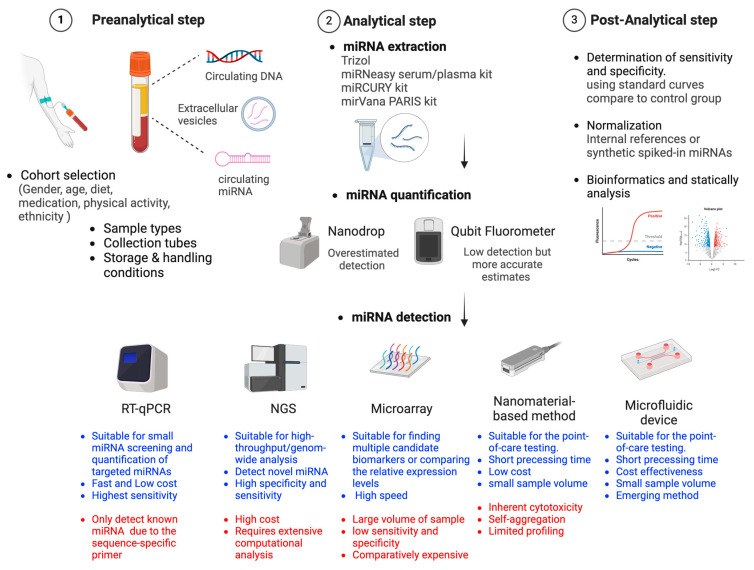
Workflow and key checkpoints for clinical applications using circulating miRNA. The main advantages (in blue) and disadvantages (in red) of miRNA detection methods are summarized.

To date, the methods commonly used for screening and profiling circulating miRNA expression are Quantitative Real-time PCR (qRT-PCR), hybridization-based technology (Microarray), and high-throughput sequencing (NGS) (Figure 1). 

qRT-PCR is one of the most widely used methods and is considered the most sensitive for quantification of miRNA expression. In general, it is a relatively simple and cost-efficient technology. qRT-PCR methods currently applied include SYBR green-based miScript (Qiagen), SYBR green-based miRCURY LNA (Exiqon), and TaqMan-based miRNA TaqMan assay (Life Technologies) [65]. These technologies have developed into qRT-PCR arrays such as custom miScript miRNA PCR array (Qiagen), Smart Chip PCR (Takara Bio, Kusatsu, Japan), and TaqMan array miRNA 384 Cards (ThermoFisher) that can simultaneously profile large sets of circulating miRNAs.

As with other methods to simultaneously measure circulating miRNAs all at once, miRNA microarray and NGS allow a large number of parallel analyses. miRNA microarray, as a semi-quantitative hybridization-based method, is useful for genome-wide circulating miRNA profiling and high-throughput detection of circulating miRNAs in body fluids [66]. Moreover, microarrays are flexible tools that can be tailored based on pathogenesis and are relatively straightforward and less expensive compared to NGS [65,66]. However, this method requires a large number of RNA samples and has technical variations in additional experimental steps, such as specific probe design and sample labeling, which implies a risk of inaccuracy. It tends to have lower specificity than qRT-PCR or NGS. Therefore, miRNA microarrays are a suitable method for finding multiple candidate biomarkers for diagnostic purposes or comparing the relative expression levels of specific miRNAs between two conditions (e.g., control vs. treatment or healthy vs. cancers). Currently, various commercial microarray platforms such as Affymetrix GeneChip miRNA array, Illumina BeadChip, Applied Microarray, and Exiqon miRCURY LNA miRNA array are available for performing wide initial screening in body fluids [65].

As a high-throughput analysis by miRNA-seq, NGS is considered a more promising method for detecting circulating miRNAs and finding novel miRNAs. This is because both qRT-PCR and microarray methods are limited in that they can only profile known or putative miRNAs, whereas NGS does not limit studies to known miRNAs. NGS does not require knowledge of target miRNAs, specific probes, or primers. In addition, NGS provides quantification of a variety of small RNAs (about 10–40 nt) with a wide dynamic range [67]. Ultra-deep sequencing of NGS makes it possible to identify mutations in miRNAs. Thus, NGS is the best method for miRNA discovery and has become the current frontline diagnostic tool for cancers. Currently, several companies offer NGS platforms, including HiSeq 2000 (Illumina), SOLiD (ABI), GS FLX+ (Roche), and Ion Torrent (Invitrogen) [67]. Although NGS can perform comprehensive and definitive analyses of miRNAs in one experiment, there are some considerations, such as high cost and availability of extensive computational infrastructure and bioinformatics support, in using NGS platforms, which may limit its applications.

To overcome the deficiencies of these conventional methods, new nanomaterial-based amplification methods such as rolling circle amplification (RCA), loop-mediated isothermal amplification (LAMP), and strand-displacement amplification (SDA) have recently been applied for miRNA detection [68]. Gold nanoparticles (AuNPs), magnetic nanoparticles, silver nanoclusters (AgNCs), and quantum dots (QDs) allow ultrasensitive detection of miRNA due to their high surface area, excellent electrical conductivity, and chemical stability [68]. Each nanomaterial-based method has its own advantages, such as simplicity, low-cost instrumentation, low sample volume, and sensitivity, but also some disadvantages, such as inherent cytotoxicity and self-aggregation that negatively affect results. Therefore, more optimized, realistic, and practical methods are still necessary for accuracy and consistency of results. Novel strategies, such as miRNA-based chips equipped with biosensors [69,70], would also be good approach to save time, reduce costs, and eliminate unnecessary steps.

Once the data are obtained from quantification of circulating miRNAs, we face the next challenge related to normalization. Normalization of miRNA data is a critical factor in accurately interpreting clinical significance. To date, the commonly used references are U6 snRNA (RNU6B), RNU19, RNU43, RNU48, U75, RNU44, 18S RNA, 5S RNA, GAPDH, miR-16, let-7a, and miR-106b [71,72,73]. Although certain miRNAs could be used as universal references for normalization of miRNA expression studies, some reference RNAs are not stable or reliable. Endogenous controls have not been standardized yet, which contributes to conflicting results. For example, RNU44, stably expressed in endometrial cancer, can serve as a reference in miRNA qPCR studies [74], but it is associated with prognosis in head and neck squamous cell carcinoma and breast cancer [75]. Circulating miR-16 can be used as a normalizing control in prostate cancer, but it is associated with prognosis for multiple myeloma [61,76]. To find endogenous miRNAs suitable as reference genes, the stability of expressed miRNAs can be evaluated using geNorm, NormFinder, and BestKeeper algorithms [77,78,79]. In addition, exogenous miRNAs such as cel-miR-39 and cel-miR-54 could be carefully considered to avoid misleading results [80,81]. In recent years, the scientific community has been making efforts to establish simple SOPs and reference genes to reduce analytical variability and increase the reproducibility of liquid biopsies [82,83]. However, determining an appropriate control sample also remains a key issue in liquid biopsies, as it is difficult in practice to ensure that the control group is indeed healthy.

### 3.2. Clinical Application of Circulating miRNAs

The first studies demonstrating the use of circulating miRNAs as a diagnostic tool for CRC were reported in 2009 [84]. In this study, upregulated miR-92 was validated in plasma collected from 90 patients with CRC by qRT-PCR a RNU6B (as a normalization signal), with a sensitivity of 89% and specificity of 70%. The miR-92 levels markedly reduced after surgical resection of tumors. The specificity of miR-92 as a diagnostic marker for CRC was further improved by combining it with miR-29a [85]. Importantly, this combination of miRNAs can discriminate early stages of cancer with small tumors from the normal control group, indicating that a panel of plasma miRNA markers offers an advantage over current CRC screening tools such as CEA and FOBT.

Further research has revealed that a broad panel of eight upregulated circulating miRNAs (miR-532-3p, miR-331, miR-195, miR-17, miR-142-3p, miR-15b, miR-532, and miR-652) can discriminate patients with CRC from those without CRC [86]. CRC screening using the panel of eight miRNAs is performed by RT-PCR using U6 as a normal reference and showed high accuracy with 88% sensitivity and 64% specificity [86]. In the case of a 5-miRNA panel (miR-331, miR-15b, miR-21, miR-142-3p, and miR-339-3p), it distinguished patients with advanced CRC from CRC patients with 91% sensitivity and 69% specificity [86]. A more recent study showed that a combination of miR-29a, miR-125b, and miR-145 has significant predictive value in early CRC detection. This panel of three miRNAs improved the predictive efficiency of CRC risk with increased sensitivity in a natural healthy population [87]. This is the greatest advantage of circulating miRNAs, showing powerful clinical potential for early detection of precancerous stages. Other studies have reported that differentially expressed patterns of circulating miRNA panels are associated with CRC liver metastasis [88,89] or lymph node metastasis [90,91]. Therefore, these trials suggest that panels combining multiple miRNAs may be a great approach for the feasible application of miRNAs with greater sensitivity and specificity, since a single miRNA may not be sufficient to be used as a clinical diagnostic and prognostic marker in a wide range of clinical trials.

Another clinical application is that circulating miRNAs can predict patient response to chemotherapeutic treatment. As an interesting example, Kudelova et al. investigated the expression of seven circulating miRNAs in paired postoperative and follow-up samples (3 months after surgery) using an miRCURY LNA miRNA RT-qPCR System to monitor patients receiving adjuvant treatment for CRC [92]. Selected circulating miRNA (miR-155-5p, miR-21-5p, miR-191-5p, miR-106a-5p, and miR-16-5p) levels were changed compared to the pre-operative state [92]. Another study showed that high levels of five miRNAs (miR-223-3p, miR-20a-5p, miR-17-5p, miR-19a-3p, and miR-7-5p) in 77 CRC patients were reduced after 5-FU-based therapy, but their levels rebounded after 3 months in responder patients [93]. Especially, the change of miR-19a-3p levels after 6 months was associated with the risk ratio for CRC recurrence and progression [93]. In the most recent phase II clinical trial (PROSPECT-R, NCT03010722), miR-652-3p was evaluated as a predictive biomarker of resistance to regorafenib, a multi-tyrosine kinase inhibitor, in colon cancer [94]. The level changes of circulating miR-652-3p were confirmed in both liquid and solid biopsies using a digital droplet PCR (ddPCR) system (Bio-Rad) and ISH. Moreover, using a PDOX (patient-derived organoids-xenotransplant) model, they demonstrated the mechanism by which elevated levels of miR-652-3p contribute to regorafenib resistance by blocking regorafenib-induced lethal apoptosis and promoting vessel co-optation [94] (Table 2).

Despite these findings, there are a few studies of circulating miRNA-relevant clinical trials (NCT04523389, NCT06342440) registered on ClinicalTrails.gov [96,97] (Table 2). Unlike cfDNAs, there are currently no FDA-approved circulating miRNA-based tools for screening CRC. To date, most of the CRC clinical trials have reported miRNA from colon or rectal tumor tissues (NCT01712958, NCT02635087, NCT03362684, and NCT02466113) [99,100,101,102] or circulating tumor cells (NCT01828918). The majority of preclinical studies and clinical trials related to circulating miRNAs in serum/plasma have focused solely on circulating miRNA characterization and their potential as diagnostic, predictive, and prognostic markers in various human cancers. Additionally, to date, there are no large population-based randomized trials, and clinical studies demonstrating the utility of miRNAs are also limited. Therefore, to achieve widespread clinical adoption, multiple challenges must be addressed, such as acceptable standard protocols, the consistency of reference miRNAs, the selection of normalization methods, the acknowledgment of ethnic diversity, and the impact of individual variability. To overcome the above hurdles, many studies are ongoing to develop and optimize methods and analyses, and several recommendations have been proposed, including using multiple reference genes along with standard spike-in miRNA concentrations for normalization, and handling samples according to guidelines [103,104,105]. Implementing these standards would allow miRNAs to be translated into clinical practice, providing personalized and precise medical strategies through minimally invasive approaches.

### 3.3. Clinical Application of Circulating Exosomal Micro-RNAs

Recent advancements in molecular diagnostics have brought circulating exosomal microRNAs (miRNAs) into the spotlight as potential noninvasive biomarkers for CRC [106]. Exosomes are small vesicles secreted by parental cells and contain a variety of biomolecules, including miRNAs, which are small noncoding RNAs that play a critical role in gene regulation [107]. In the context of cancer, these exosomal miRNAs are released into the circulation and reflect the pathological state of the tumor, making them a valuable source for biomarker discovery [108]. Several studies have shown that exosomal miRNA could be useful in prognostic prediction approaches. Distant metastasis from colon cancer to the liver is the major cause of colon cancer-related mortality. High levels of exosomal miR-141-3p and miR-375 derived from plasma were correlated with liver metastatic progression in rectal cancer, affecting patients’ immune activity [109]. Many other exosomal miRNAs, such as miR-548c-5p, miR-17-5p, and miR-92a-3p have been reported to contribute to liver metastasis via blood circulation [110,111]. 

Recent studies have indicated that specific exosomal miRNAs are differentially expressed in CRC patients compared to healthy controls [112]. For example, serum exosomal levels of miRNAs such as let-7a, miR-1229, miR-1246, miR-150, miR-21, miR-223, and miR-23a were found to be significantly higher in patients, particularly in early-stage CRC [106]. These levels were observed to decrease postsurgically, suggesting a direct association with tumor burden. The diagnostic potential of these miRNAs has been explored through microarray analyses and validated by quantitative real-time RT-PCR [106]. These studies underscore the high sensitivity of selected exosomal miRNAs, surpassing traditional tumor markers like CA19-9 and CEA in some cases. Moreover, the expression profiles of these miRNAs correlate with patient survival rates, indicating their value not only in diagnosis but also in prognosis. The variability in miRNA expression post-tumor resection highlights the possibility of using these biomarkers for monitoring disease progression and treatment efficacy. The translation of these findings into clinical practice could revolutionize CRC management by enabling early detection, real-time monitoring of treatment response, and possibly guiding therapy choices based on miRNA profiles. The noninvasive nature of blood-based tests offers a patient-friendly alternative to conventional diagnostic methods such as colonoscopy. 

While the promise is substantial, the clinical application of circulating exosomal miRNAs still faces challenges. To date, some clinical trials (NCT04523389, NCT06342440, and NCT04227886) [96,97,98] have aimed to identify specific exosomal miRNAs for CRC (Table 2). If further studies address several hurdles, including standardization of exosome isolation and reproducibility, these clinical trials may help develop the effectiveness of blood tests with exosomal miRNAs for CRC screening. Furthermore, understanding the mechanistic role of these miRNAs in CRC pathogenesis could open new avenues for targeted therapies, potentially leading to miRNA-based therapeutic interventions.

### 3.4. Theranostic Values of Circulating Micro-RNAs

Circulating miRNAs have garnered interest as potential theranostic biomarkers due to their stability in body fluids and their regulatory roles in gene expression. As diagnostic circulating miRNAs are generally involved in various biological processes and disease mechanisms, they are promising candidates for disease progress, treatment predictor, and treatment response markers. The identification of specific miRNAs in blood that correlate with CRC presence and progression can significantly enhance early detection and personalized treatment strategies. In addition, diagnostic values of circulating miRNAs can be used for drug discovery and therapeutic targets. 

Currently, several clinical trials evaluating circulating miRNAs as treatment predictors and response markers for CRC are listed at ClinicalTrials.gov [94,95,98]. A recent active clinical trial is investigating the correlation between changes in circulating miRNA levels and neoadjuvant treatment, surgery, and adjuvant chemotherapy in patients with colorectal cancer [95]. It uses NanoString technology to detect specific miRNAs among multiple targets present in the same sample. The description of this study informs that differentially expressed circulating miRNAs will predict the tumor response to therapies. We anticipate that miRNA level assessment at all stages of treatment may be useful for evaluating the effectiveness of neoadjuvant treatment and monitoring pathologic complete response (pCR) with minimal residual tissue after surgical resection. As another preclinical study, miR-21 has been widely studied for its role in promoting tumor growth and its elevated levels in the plasma of CRC patients, making it a valuable marker for CRC progression and a target for therapeutic intervention and response [113]. 

Certain miRNA profiles can predict disease recurrence and patient survival, aiding in the stratification of patients based on risk and guiding treatment decisions. Therapeutically, targeting dysregulated miRNAs in CRC has shown potential in preclinical studies, with miRNA mimics and inhibitors being developed to restore normal gene expression and inhibit tumor progression. Overall, the theranostic potential of circulating miRNAs in CRC continues to be an area of active research, promising advancements in early detection, prognosis, and personalized therapy [114].

## 4. Conclusions

Circulating biomarkers such as circulating DNAs and miRNAs hold significant promise for the noninvasive diagnosis and prognosis of CRC. As research advances, these biomarkers stand at the forefront of a paradigm shift towards precision oncology, heralding a new era of cancer management where blood-based biomarkers guide clinical decision-making, improving patient outcomes through personalized medicine. 

Challenges in using circulating nucleic acids as biomarkers include their low abundance, especially in early-stage cancers, lack of standardized protocols, difficulties in normalization, and issues with specificity. Technical variations in extraction and analysis methods, the high costs of advanced technologies, and the complexity of data interpretation also pose significant hurdles. Moreover, translating promising biomarkers from research to clinically validated tests remain challenging.

To overcome these obstacles, future directions focus on developing more sensitive detection methods, including advanced PCR techniques and nanotechnology-based approaches. Standardizing protocols for sample handling and analysis is crucial. Efforts should be made to identify robust reference genes for normalization and to use multi-marker panels to improve specificity and sensitivity. Integrating circulating nucleic acid markers with other biomarker types could enhance accuracy. Large-scale clinical trials are needed to validate promising biomarkers in diverse populations. Prioritizing research on early-stage cancer detection and developing user-friendly, cost-effective point-of-care devices is also important. Finally, exploring novel circulating RNAs and improving bioinformatics tools will be key to advancing the field of liquid biopsy for cancer detection and monitoring.

## Figures and Tables

**Table 1:** Circulating DNA-based clinical trials in colorectal cancer.

Method	Sample Type	Cohort Size	Main Characteristics	Clinical Trial	Ref.
OncoBEAM RAS CRC kit and Guardant360	Plasma	Metastatic CRC patients with RAS/BRAF V600E wild-type tumor resistant to anti-EGFR therapy (REMARRY; n = 183, PURSUIT; n = 50)	RAS ctDNA status as a predictive marker for determining anti-EGFR treatment eligibility and RAS-negative ctDNA as an assessment for the efficacy and safety of therapy with panitumumab plus irinotecan	REMARRY (UMIN000036424) and PURSUIT study (jRCTs031190096)	[30]
ddPCR	Plasma	Patients resected for CRC and treated with adjuvant chemotherapy (n = 254)	Detectable ctDNA level as a diagnostic marker for early detection of CRC recurrence and determination of treatment eligibility	IMPROVE-IT2 (NCT04084249)	[18]
qRT-PCR: SEPT9 real-time PCR	Plasma	CRC patients (n = 100) and noncancer group (n = 175) in training study, CRC patients (n = 90) and noncancer group (n = 155) in blinded testing study	Methylated SEPT9 as a diagnostic marker for CRC screening	PRESEPT (NCT00855348, NCT02540850)	[31]
NGS; FoundationACT™, Illumina Hi-Seq comprehensive genomic profiling	Plasma	96 CRC patients (II, III, IV stage)	KRAS, NRAS, BRAF ctDNA as a diagnostic marker and predictive marker for guiding therapy in the late stage	NCT02620527	[23]
NGS; Guardant LUNAR-2™	Blood	200 clinical trial sites; 13% black, 15% Hispanic and 7% Asian American, 45–84 age without prior history cancer (n = 10,000)	ctDNA as an early detector for CRC screening with 96% sensitivity, 94% specificity	ECLIPSE (NCT04136002)	[32]
NGS: Guardant LUNAR-1™	Blood	Stage II/III patients with resected stage II (n = 1408)	ctDNA as a predictive marker for adjuvant chemotherapy and minimal residual disease (MRD) determination	COBRA (NCT04068103)	[33]
NGS; FoundationOne Liquid	Plasma	Metastatic CRC with RAS/BRAF wild type tumor (n = 200)	RAS/BRAF ctDNA status as a predictive marker of response to FOLFOX therapy in patients resistant to anti-EGFR therapy	CAPRI II GOIM (NCT05312398)	[34]
NGS; Guardant Reveal	Plasma	Patients resected for stage II/III CRC (n = 1621)	ctDNA presence as a biomarker to guide treatment decisions, with the potential to reduce adjuvant chemotherapy related toxicities	TRACC (NCT04050345)	[35]
PCR NGS: Signatera test	Plasma	Stage I–IV patients (n = 2000) who have undergone surgery	Detectable ctDNA level as a diagnostic and prognostic marker for detection of CRC recurrence and determination of adjuvant treatment	BESPOKE (NCT04264702)	[36]
PCR NGS: Signatera MRD blood test	Plasma	2500 CRC patients	Positive/negative ctDNA and 16 specific somatic variants as a predictive marker for monitoring MRD and the effectiveness of adjuvant chemotherapy in CRC with surgery	CIRCULATE-Japan ((UMIN000039205)	[37,38]

**Table 2:** Circulating RNA-based clinical trials in colorectal cancer.

Method	Sample Type	Cohort Size	Main Characteristics	Clinical Trial	Status	Ref.
NanoString nCounter/ddPCR	Blood (plasma)	Patients treated with regorafenib for RAS mutant metastatic CRC (n = 40)	miR-652-3p as a predictive marker of resistance and response to regorafenib monotherapy	PROSPECT-R (NCT03010722)	Completed	[94]
NanoString	Blood (plasma)	Patients diagnosed with adenocarcinoma of the rectum (n = 200)	miRNA levels as a predictive marker for monitoring MRD and a putative marker of the response to neoadjuvant treatment	NCT03962088	Recruiting	[95]
Unknown	Blood	Patients diagnosed with colorectal cancer between 2008 and 2012 (n = 172)	Exosome miRNA as an early diagnostic and prognostic marker for CRC	NCT04523389	Unknown	[96]
qPCR analysis	Blood	Patients with advanced Adenomas and CRC (n = 1000)	Exosome miRNA as an early diagnostic marker for Advanced Adenomas and Colorectal Cancer	NCT06342440	Recruiting	[97]
Unknown	Blood	Patients resected for CRC and treated with adjuvant chemotherapy (n = 250)	Exosome miRNA as a predictive Biomarker of Neoadjuvant Chemoradiotherapy for Rectal Cancer	NCT04227886	Unknown	[98]
Unknown	Tissue	Patients underwent colon resection (n = 100)	miRNA levels as a prognostic or therapeutic marker for CRC	NCT01712958	Unknown	[99]
q-RT-PCR	Tissue	Patients diagnosed with stage II colon cancer (n = 630)	miR-21, miR-20a-5p, miR-10a-3p, miR-106b-5p, miR-143-5p, and miR-215 as a predictive marker for stage II CRC treated with chemotherapy	NCT02635087	Recruiting	[100]
RT-qPCR	Tissue	Resected stage III CRC patients to receive adjuvant treatment with either FOLFOX-4 plus cetuximab or FLOFOX-4 alone (n = 1808)	miR-31-3p and miR-31-5p as a prognostic and predictive marker for outcome and benefit to anti-EGFR therapy	NCT03362684	Completed	[101]
RT-qPCR	Tissue	Patients diagnosed with stage CRC (n = 430)	miR-21, miR-20a-5p, miR-103a-3p, miR-106b-5p, miR-143-5p, and miR-215 as a predictive marker for determination of adjuvant treatment	NCT02466113	Active, not recruiting	[102]
